# Does the Bacillus Abortus Infect Man?

**Published:** 1915-11

**Authors:** 


**Does the Bacillus Abortus Infect Man?** Three papers in the American Journal of Diseases of Children (Vol. 10, No. 3), are devoted to this subject. The bacillus abortus, proved to be the specific cause of contagious abortion in cattle by Bang of Copenhagen in 1906, produces in various laboratory animals certain pathological changes microscopically identical with those caused by tubercle bacilli, and certain other changes which involve, spleen, liver and kidneys.

Sedwick and Larson of Minneapolis present their second contribution to this subject. They tested the blood of many children using a complement fixation test with the bacillus abortus as antigen. They found no positive reactions in babies fed on breast milk alone. When cow's milk was included in the diet a certain number of positive reactions appeared. By further study they were able to show that babies whose milk supply came from herds kept under ideal conditions and not infected with, or exposed to, contagious abortion did not react, while infants fed raw milk from unselected dairies frequently reacted positively.

Sedgwick and Larson realize that their work is not entirely conclusive, but believe that the possibility of infection justifies them in insisting on breast feeding or at least rigid inspection or adequate heating of all milk used in infant feeding.

A brief paper by Ramsey of St. Paul records, without comment, the discovery of seven positive reactions in an orthopedic hospital. The complement fixation tests were made by Larson of Minneapolis.

Nicoll and Pratt report extensive work done in the laboratories of the Department of Health in New York City. They note that isolation of the bacillus abortus from human tissue has been reported only in a single case. Even here infection was not proved, as the organism occurred on the tonsil and its presence may have been accidental. They recognize the value of various investigations, specially those of Sedgwick and Larson, but believe that ingestion of the bacillus may be responsible for many complement fixations or agglutination reactions. In this connection they call attention to the positive serum reactions obtainable by feeding guinea pigs with typhoid bacilli. In conclusion, results with agglutination and inoculation experiments were obtained after a considerable series of cases were investigated. On the whole they are inclined to wait for successful isolation of the bacillus abortus from human tissue before regarding other tests as more than suggestive.

